# Impact of an eHealth Smartphone App on the Mental Health of Patients With Psoriasis: Prospective Randomized Controlled Intervention Study

**DOI:** 10.2196/28149

**Published:** 2021-10-25

**Authors:** Lena Domogalla, Alena Beck, Theresa Schulze-Hagen, Raphael Herr, Johannes Benecke, Astrid Schmieder

**Affiliations:** 1 Department of Dermatology University Medical Center Mannheim Heidelberg University Mannheim Germany; 2 Mannheim Institute of Public Health, Social and Preventive Medicine Medical Faculty Mannheim Heidelberg University Mannheim Germany; 3 Department of Dermatology University Hospital Würzburg Würzburg Germany

**Keywords:** psoriasis, eHealth, mHealth, telemedicine, teledermatology, patient educational program, disease management, smartphone app, mental health, mobile phone

## Abstract

**Background:**

Psoriasis has a negative impact on patients’ physical and mental health and can lead to anxiety and depression. Disease management strategies, including educational programs and eHealth devices, have been shown to improve health care for several chronic diseases. However, such disease management strategies are lacking in the routine care of patients with psoriasis.

**Objective:**

This study aims to study the impact of a novel intervention that combines an educational program with a disease management smartphone app on the mental health of patients with psoriasis.

**Methods:**

Patients with psoriasis in the intervention group received an educational program; attended visits on weeks 0, 12, 24, 36, and 60; and had access to the study app. Patients in the control group only attended the visits. The primary endpoint was a significant reduction of scores on the Hospital Anxiety and Depression Scale (HADS). Secondary end points were reductions in Dermatology Life Quality Index score, Psoriasis Area and Severity Index score, pruritus, and pain, as well as improvements in mood and daily activities. In addition, modulating effects of sex, age, disease duration, and app use frequency were evaluated.

**Results:**

A total of 107 patients were included in the study and randomized into the control group (53/107, 49.5%) or intervention group (54/107, 50.5%). Approximately 71.9% (77/107) of the patients completed the study. A significant reduction in HADS-Depression (HADS-D) in the intervention group was found at weeks 12 (*P*=.04) and 24 (*P*=.005) but not at weeks 36 (*P*=.12) and 60 (*P*=.32). Patient stratification according to app use frequency showed a significant improvement in HADS-D score at weeks 36 (*P*=.004) and 60 (*P*=.04) and in HADS-Anxiety (HADS-A) score at weeks 36 (*P=.*04) and 60 (*P*=.05) in the group using the app less than once every 5 weeks. However, in patients using the app more than once every 5 weeks, no significant reduction in HADS-D (*P*=.84) or HADS-A (*P*=.20) score was observed over the 60-week study period compared with that observed in patients in the control group. All findings were independent of sex, age, and disease duration.

**Conclusions:**

These findings support the use of a disease management smartphone app as a valid tool to achieve long-term improvement in the mental health of patients with psoriasis if it is not used too frequently. Further studies are needed to analyze the newly observed influence of app use frequency.

**Trial Registration:**

Deutsches Register Klinischer Studien DRKS00020755; https://tinyurl.com/nyzjyvvk

## Introduction

### Background

Psoriasis is a common, chronic inflammatory skin disease that affects approximately 2.53% of the German population and has a significant impact on physical and mental health [1-3]. In addition to physical symptoms such as pain and itching, patients with psoriasis report stigmatization, shame, lack of self-confidence, depression, and anxiety as major impairments [4-6]. Moreover, low therapy adherence in patients and consequent poor disease control results from a lack of knowledge regarding the disease [7].

For several different chronic diseases, educational programs are part of routine care because they improve patients’ self-management skills and reduce disease activity [8]. A routine educational program is not in place to cater to patients who wish to be better informed about their illness and more involved in therapy decisions [7-9]. The pilot study conducted by Bubak et al [10] showed that an educational program for patients with psoriasis alone leads to a significant improvement in the knowledge of their disease and to an amelioration of general health, but not to an improvement in mental health. Several other studies have reported educational programs as valid tools to improve self-expertise, therapy adherence, and quality of life in patients with psoriasis; encourage lifestyle changes; and reduce disease severity [11-14].

Another possibility to improve patient care is using eHealth devices. Since 2019, Germany has allowed the prescription of scientifically validated digital health apps. In several studies, eHealth devices have shown positive effects on common chronic diseases such as diabetes, hypertension, chronic heart failure, and asthma [15-18]. For psoriasis, the data are still limited; however, digital care seems to be as safe and effective as regular in-person care [19-21]. So far, no studies that evaluate smartphone apps for long-term disease management in psoriasis exist.

### Objectives

In this study, we combined the educational program already presented by Bubak et al [10] with a disease management smartphone app, generating a new possibility to strengthen patients’ self-management, reduce disease burden, and build a more trusting doctor-patient relationship. In a first interim analysis of this study, after 6 months, a significant improvement in depressive symptoms was found in the intervention group receiving the educational program and the study app. In addition, a clear impact of the app use frequency was assessed. Patients using the app <20% (ie, less than once every 5 weeks) had a significantly stronger reduction in depressive and anxiety symptoms [22]. This study investigates the long-term effects over 60 weeks of a patient educational program combined with a psoriasis app on the mental health of patients with psoriasis and examines the mediating effect of app use frequency more closely.

## Methods

### Study Design and Patients

This intervention study was undertaken at the Department of Dermatology, Venereology, and Allergology at the University Medical Center Mannheim, Germany, between January 2018 and June 2020. Patients were recruited at the outpatient clinic, where they were asked to participate in the study while attending their regular doctors’ appointments. Eligible patients were aged 18 to 75 years, able to provide written informed consent, had a physician-confirmed diagnosis of moderate-to-severe psoriasis (defined as Psoriasis Area and Severity Index [PASI] score >10; body surface area >10; Dermatology Life Quality Index [DLQI] score >10; psoriatic involvement of the scalp, palms, soles, and genital area; or psoriatic nail involvement of at least 2 nails). The exclusion criteria were the inability to provide written informed consent and no access to a smartphone.

The study was conducted in accordance with the Declaration of Helsinki and approved by the Medical Ethics Committee of the Medical Faculty Mannheim, Heidelberg University (ethics approval 2017-655N-MA). The trial is registered at Deutsches Register Klinische Studien (registration number: DRKS00020755). Written informed consent was provided by each patient before their participation in the study. Eligible participants were randomized into an intervention or control group in a 1:1 ratio. For this purpose, sealed envelopes labeled *intervention group* or *control group* were randomly assigned to each patient during the baseline visit.

**Figure 1:**
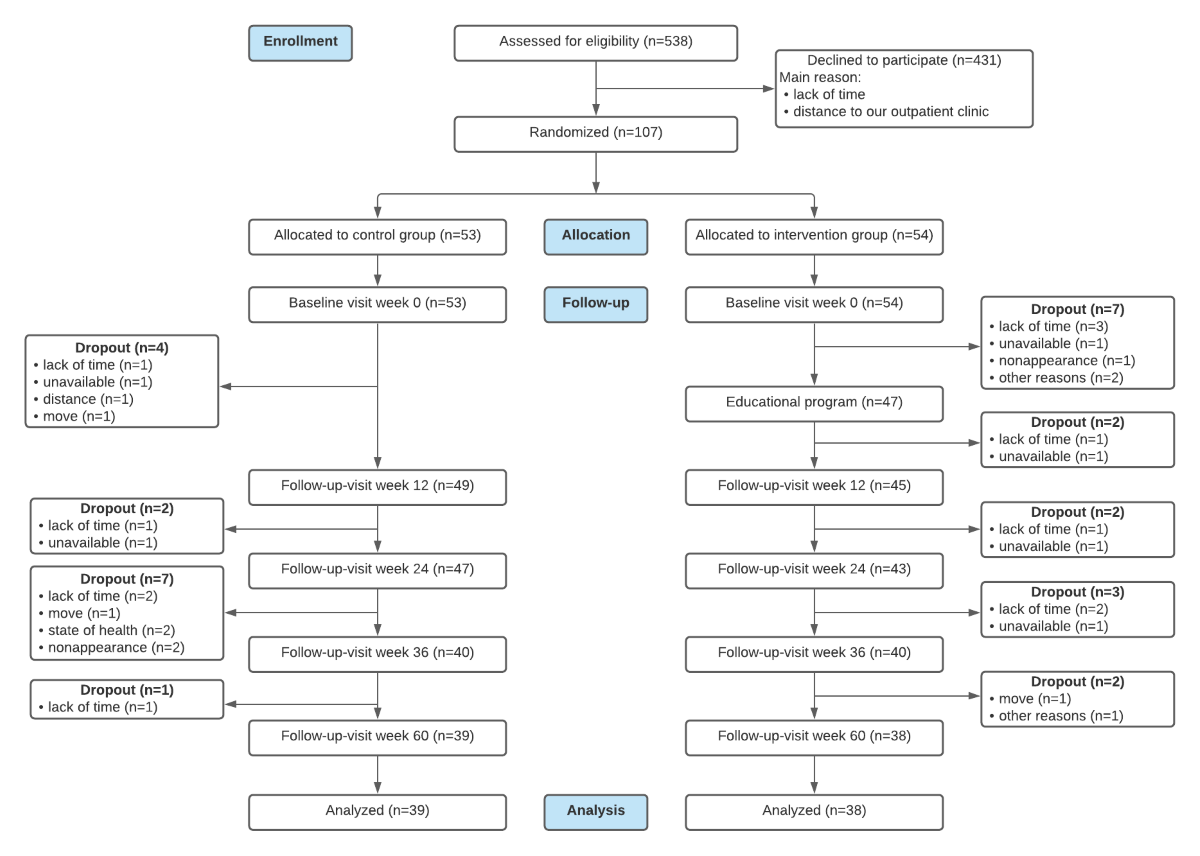
Consolidated Standards of Reporting Trials (CONSORT) flow diagram of the study cohort.

The control group (n=53) started the 60-week study period with their baseline visit in week 0, when they indicated sociodemographic and psoriasis-related data. In addition, the PASI (range 0-72), DLQI (range 0-30), Hospital Anxiety and Depression Scale–Anxiety/Depression (HADS-A/D; range 0-21 for anxiety and depression each), and numeric rating scales for skin pain and pruritus (range 0-10 each) were assessed. Furthermore, the patients indicated how much psoriasis negatively affected their mood and daily activity levels during the past weeks on a Likert scale (range 0-3 each; 0=not at all, 1=a little, 2=fairly, and 3=very). In-person follow-up visits with the same assessments took place at weeks 12, 24, and 36 and the final visit at week 60 (Figure 1).

The intervention group (n=54) participated in the same baseline and follow-up visits as the control group. In addition, they attended a 2-hour-long educational program on the topic of psoriasis, which was held by specialists in dermatology (AS and JB) after the baseline visit (Figure 1). The educational program was held in a group setting and included information on psoriasis pathogenesis, therapy options, and comorbidities (Multimedia Appendix 1) [10]. Each participant received a personal anonymized access code and an introduction to our psoriasis monitoring app DermaScope Mobile (Multimedia Appendices 2 and 3). For 60 weeks, the patients were able to document their psoriasis regularly by photodocumentation of their skin and answering health questionnaires regarding their quality of life, mood, activity, pain, and pruritus via the app. App use frequency for adding new data was limited to once a week. Furthermore, the patients could contact specialized dermatologists unrestrictedly (AS and JB) via a chat feature within the app. Consort eHealth checklist can be found in the Multimedia Appendix 4.

### Outcomes

The primary end point was a significant reduction in the HADS-D/-A in the intervention group at the follow-up visits compared with the control group. Secondary endpoints were the reduction in the PASI and numeric rating scale for pruritus and pain and an improvement in the DLQI and mood and daily activity Likert Scales at the follow-up. Modulating effects of sex, age, disease duration, and app use frequency were evaluated for each time point.

### Statistical Analysis

Linear panel data regression analyses estimated the trajectories of the outcomes. Random-effect regressions determined the main and interaction effects of group membership (intervention vs control group; interaction *week x group* in Multimedia Appendix 5) and visit time point (interaction *week* in Multimedia Appendix 5) on HADS-D, HADS-A, DLQI, mood, daily activity, PASI, pruritus, and pain (Multimedia Appendix 5). Two models of adjustment were calculated. One was unadjusted, whereas the other was adjusted for sex, age, and disease duration. In additional analyses, the effects of the app use frequency over 60 weeks were included (group membership: control vs <20% vs ≥20% use; interaction *week x group* in Multimedia Appendix 6). The chosen cutoff of 20% equals one-time use every 5 weeks. Variables were transformed to approach normal distribution (power transform square root of HADS-D and HADS-A, log10 of DLQI, and square of mood; no transformation of daily activity, pruritus, and pain). All statistical analyses were performed using STATA SE 14 (StataCorp LLC). A statistically significant difference was assumed at *P*≤.05.

## Results

### Study Cohort

This study was conducted between January 2018 and June 2020. Of the 538 patients assessed, 431 (80.1%) were found to be ineligible because of lack of time, distance to our outpatient clinic, and no access to a smartphone. Of the 538 patients, 107 (19.9%) patients joined the study. After randomization to the control (53/107, 49.5%) or intervention (54/107, 50.5) groups, all patients participated in the baseline visit. After the baseline visit, 10.3% (11/107) of patients discontinued the study (control group: 4/11, 36%; intervention group: 7/11, 64%). The remaining 87% (47/54) of patients in the intervention group attended the educational program. Until week 60, 20% (10/49) of patients from the control group and 19% (9/47) of patients from the intervention group discontinued the study (Figure 1). The most common reasons for withdrawal were lack of time (12/107, 11.2%) and unavailability or nonappearance (10/107, 9.3%). Other reasons included distance to our outpatient clinic or relocating (4/107, 3.7%), poor health status (2/107, 1.9%), and other reasons (3/107, 2.8%). Of the 107 patients, 77 (72.0%) completed the study; 74% (39/53) from the control group and 70.4% (38/54) from the intervention group completed the study. Over the entire study period, the demographic, socioeconomic, and psoriasis-related characteristics were well balanced between the control and intervention groups (Tables 1 and 2), except for a slightly older control group (*P*=.04). At week 60, 9% (7/77) of patients were treated with disease-modifying antirheumatic drugs, and 64% (49/77) were treated with biologics. Of the 77 patients, 16 (21%) received topical or phototherapy, 2 (3%) received other systemic drugs, and 3 (4%) were not treated with any antipsoriatic therapy.

**Table 1:** Characteristics of the study cohort at weeks 0 (N=107).

Characteristic at week 0^a^			Overall		Control group (n=53)		Intervention group (n=54)		*P* value
**Sex, n (%)**									
	Female	42 (39.3)		23 (43)		19 (35)		.38^b^	
	Male	65 (60.7)		30 (57)		35 (65)		.38^b^	
**Age (years)**									
	Mean (SD)	49.1 (12.1)		51.7 (11.8)		46.5 (11.9)		.03^c^	
	Median (IQR)	51.0 (42-57)		53.0 (49-59)		48.0 (36-54)		.03^c^	
**BMI (kg/m^2^**)									
	Mean (SD)	29.1 (5.7)		29.3 (6.1)		28.9 (5.3)		.72^b^	
	Median (IQR)	28.3 (24.8-32.5)		28.3 (24.7-32.2)		28.2 (24.9-32.5)		.72^b^	
**Alcohol (days/week)**									
	Mean (SD)	1.1 (1.5)		1.1 (1.5)		1.2 (1.5)		.68^c^	
	Median (IQR)	1.0 (0-2)		0.0 (0-2)		1.0 (0-1.5)		.68^c^	
Smoker, n (%)			35 (32.7)		20 (38)		15 (28)		.27^b^
**Duration of psoriasis (years)**									
	Mean (SD)	18.9 (14.6)		20.4 (15.5)		17.5 (13.7)		.31^c^	
	Median (IQR)	15.0 (6-28)		18.0 (7-34)		13.0 (6-26)		.31^c^	
Psoriatic arthritis, n (%)			46 (43.0)		24 (45)		22 (42)		.76^b^
**Antipsoriatic therapy, n (%)**									
	Topical or UV therapy	31 (29.0)		14 (26)		17 (31)		.43^b^	
	DMARDS^d^	17 (15.9)		6 (11)		11 (20)		.43^b^	
	Others	1 (0.9)		1 (2)		0 (0)		.43^b^	
	Biologicals	51 (47.7)		29 (55)		22 (41)		.43^b^	
	No therapy	7 (6.5)		3 (6)		4 (7)		.43^b^	
**HADS-D^e^** **(range 0-21)**									
	Mean (SD)	5.3 (4.7)		5.2 (5.0)		5.3 (4.3)		.94^c^	
	Median (IQR)	4.0 (1-8)		3.0 (1-8)		4.5 (2-8)		.94^c^	
**HADS-A^f^** **(range 0-21)**									
	Mean (SD)	6.9 (4.4)		7.0 (4.9)		6.7 (3.8)		.66^c^	
	Median (IQR)	6.0 (3-10)		6.0 (3-10)		6.0 (4-9)		.66^c^	
**DLQI^g^** **(range 0-30)**									
	Mean (SD)	8.2 (8.0)		8.5 (8.5)		7.9 (7.6)		.69^c^	
	Median (IQR)	5.0 (2-13)		5.0 (3-12)		5.0 (2-13)		.69^c^	
**Mood (range 0-3)**									
	Mean (SD)	1.2 (1.2)		1.2 (1.2)		1.2 (1.2)		.92^c^	
	Median (IQR)	1.0 (0-2)		1.0 (0-2)		1.0 (0-2)		.92^c^	
**Daily activity (range 0-3)**									
	Mean (SD)	1.3 (1.1)		1.4 (1.1)		1.1 (1.1)		.30^c^	
	Median (IQR)	1.0 (0-2)		1.0 (0-2)		1.0 (0-2)		.30^c^	
**PASI^h^** **(range 0-72)**									
	Mean (SD)	5.1 (5.2)		5.1 (5.1)		5.0 (5.4)		.98^c^	
	Median (IQR)	3.0 (1.4-7.6)		3.6 (1.4-7.2)		3.0 (1.5-7.6)		.98^c^	
**Pain (range 0-10)^i^**									
	Mean (SD)	2.0 (2.4)		2.2 (2.7)		1.9 (2.2)		.53^c^	
	Median (IQR)	1.0 (0-4)		1.0 (0-3)		1.0 (0-4)		.53^c^	
**Pruritus (range 0-10)**									
	Mean (SD)	3.0 (2.7)		3.2 (3.1)		2.8 (2.2)		.35^c^	
	Median (IQR)	2.0 (1-5)		2.0 (1-5)		2.0 (1-4)		.35^c^	
**App use frequency, n (%)**									
	<20%	20 (18.7)		0 (0)		20 (37)^j^		<.001^b^	
	≥20%	20 (18.7)		0 (0)		20 (37)^j^		<.001^b^	
	No app use	67 (62.6)		53 (100)		14 (26)^j^		<.001^b^	

^a^Data for sex, age, BMI, smoking, alcohol consumption, psoriasis duration, psoriasis arthritis, and therapy were collected at week 0 only.

^b^Categorical variables were analyzed using the chi-square test.

^c^Continuous variables were analyzed using the *t* test.

^d^DMARDs: disease-modifying antirheumatic drugs.

^e^HADS-D: Hospital Anxiety and Depression Scale–Depression.

^f^HADS-A: Hospital Anxiety and Depression Scale–Anxiety.

^g^DLQI: Dermatology Life Quality Index.

^h^PASI: Psoriasis Area and Severity Index.

^i^For 1 patient, data for weight and height were missing.

^j^App use frequency for week 0 was identified retrospectively in weeks 0 to 12. From the intervention group, 14 patients did not download the app at all or dropped out of the study. Therefore, they could not be divided into app use frequency subgroups.

**Table 2:** Characteristics of the study cohort at weeks 60 (N=77).

Characteristics week 60			Overall (n=77)		Control group (n=39)		Intervention group (n=38)		*P* value
**Sex, n (%)**									
	Female	28 (36)		13 (33)		15 (39)		.58^a^	
	Male	49 (64)		26 (67)		23 (61)		.58^a^	
**Age (years)**									
	Mean (SD)	49.6 (11.7)		52.3 (10.5)		46.9 (12.3)		.04^b^	
	Median (IQR)	52.0 (45-57.0)		54.0 (50-59)		48.0 (39-54)		.04^b^	
**BMI (kg/m^2^)**									
	Mean (SD)	28.3 (5.6)		28.9 (6.3)		27.6 (4.7)		.33^b^	
	Median (IQR)	26.6 (24.5-31.7)		26.6 (24.5-31.2)		26.0 (24.2-32.5)		.33^b^	
**Alcohol (days/week)**									
	Mean (SD)	1.3 (1.6)		1.3 (1.6)		1.3 (1.7)		.99^b^	
	Median (IQR)	1.0 (0-2.5)		1.0 (0-3)		1.0 (0-2)		.99^b^	
Smoker n (%)			26 (34)		16 (41)		10 (27)		.17^a^
**Duration of psoriasis (years)**									
	Mean (SD)	19.2 (14.8)		21.2 (16.1)		17.1 (13.1)		.22^b^	
	Median (IQR)	15.0 (6-28)		18.0 (7-37)		13.5 (6-24)		.22^b^	
Psoriatic arthritis, n (%)			35 (45.5)		20 (51.3)		15 (39.5)		.35^a^
**Antipsoriatic therapy, n (%)**									
	Topical or UV therapy	16 (21)		5 (13)		11 (29)		.07^a^	
	DMARDS^c^	7 (9)		3 (8)		4 (11)		.07^a^	
	Others	2 (3)		0 (0)		2 (5)		.07^a^	
	Biologicals	49 (64)		28 (72)		21 (55)		.07^a^	
	No therapy	3 (4)		3 (8)		0 (0)		.07^a^	
**HADS-D (range 0-21)^d^**									
	Mean (SD)	4.0 (4.4)		4.1 (4.2)		4.0 (4.5)		.94^b^	
	Median (IQR)	2.0 (0-7)		3.0 (0-7)		2.0 (0-7)		.94^b^	
**HADS-A (range 0-21)^e^**									
	Mean (SD)	5.5 (3.8)		6.0 (4.0)		4.9 (3.5)		.22^b^	
	Median (IQR)	5.0 (2-8)		6.0 (2-8)		4.0 (2-7)		.22^b^	
**DLQI (range 0-30)^f^**									
	Mean (SD)	4.1 (5.5)		3.7 (4.1)		4.4 (5.5)		.56^b^	
	Median (IQR)	1.0 (1-7)		1.0 (1-7)		1.0 (1-8)		.56^b^	
**Mood (range 0-3)**									
	Mean (SD)	0.7 (0.7)		0.7 (0.7)		0.6 (0.8)		.71^b^	
	Median (IQR)	1.0 (0-1)		1.0 (0-1)		0.5 (0-1)		.71^b^	
**Daily activity (range 0-3)**									
	Mean (SD)	0.6 (0.7)		0.6 (0.7)		0.5 (0.7)		.59^b^	
	Median (IQR)	0.0 (0-1)		0.0 (0-1)		0.0 (0-1)		.59^b^	
**PASI (range 0-72)^g^**									
	Mean (SD)	2.6 (3.0)		2.6 (2.9)		2.7 (3.3)		.90^b^	
	Median (IQR)	1.5 (0.4-3.4)		1.7 (0-3.4)		1.4 (0.6-3.4)		.90^b^	
**Pain (range 0-10)^h^**									
	Mean (SD)	1.5 (2.5)		2.0 (2.8)		1.0 (2.0)		.10^b^	
	Median (IQR)	0.0 (0-2.5)		0.0 (0-3)		0.0 (0-1)		.10^b^	
**Pruritus (range 0-10)**									
	Mean (SD)	1.8 (2.1)		1.8 (2.1)		1.8 (2.2)		.95^b^	
	Median (IQR)	1.0 (0-3)		1.0 (0-3)		1.0 (0-3)		.95^b^	
**App use frequency, n (%)**									
	<20%	15 (20)		0 (0)		15 (39)		<.001^a^	
	≥20%	18 (23)		0 (0)		18 (47)		<.001^a^	
	No app use	44 (57)		39 (100)		5 (13)		<.001^a^	

^a^Categorical variables were analyzed using the chi-square test.

^b^Continuous variables were analyzed using the *t* test.

^c^DMARDs: disease-modifying antirheumatic drugs.

^d^HADS-D: Hospital Anxiety and Depression Scale–Depression.

^e^HADS-A: Hospital Anxiety and Depression Scale–Anxiety.

^f^DLQI: Dermatology Life Quality Index.

^g^PASI: Psoriasis Area and Severity Index.

^h^For 1 patient, data for pain were missing at week 60.

### Outcomes

**Figure 2:**
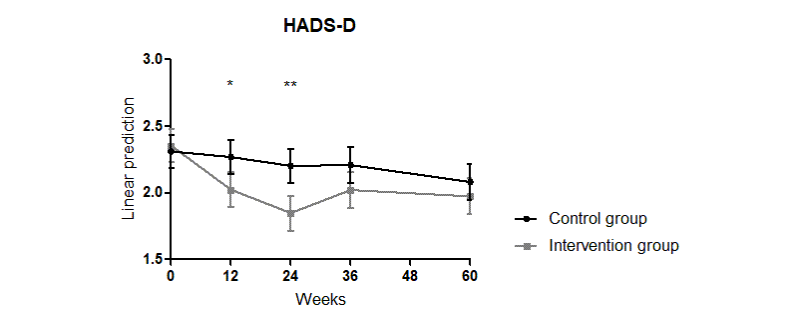
Significant improvement of the HADS-D score in patients with psoriasis by combining an educational program with a psoriasis app over 24 weeks. HADS-D: Hospital Anxiety and Depression Scale–Depression. *P≤.05, **P≤.01.

The significant reduction in the HADS-D in the intervention group compared with that in the control group found at weeks 12 and 24 did not persist in weeks 36 and 60 (interaction week x intervention: week 12: coefficient=–0.289, *P*=.04; week 24: coefficient=–0.397, *P*=.005; week 36: coefficient=0.231, *P*=.12; week 60: coefficient=0.15, *P*=.32; Model 0 in Multimedia Appendix 5 and Figure 2). The HADS-D score was significantly lower in both groups at week 60 compared with the score at the baseline visit (interaction week 60: coefficient=0.23, *P*=.03; Model 0 in Multimedia Appendix 5).

**Figure 3:**
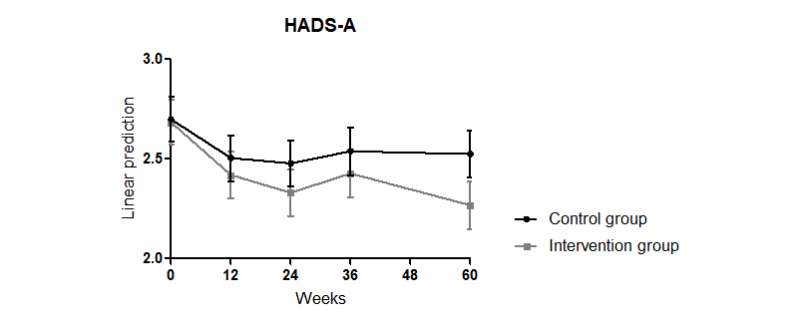
Effects of an educational program combined with a disease management psoriasis app on the HADS-D score over 60 weeks. HADS-D: Hospital Anxiety and Depression Scale–Depression.

The improvement found in the HADS-A in all patients until week 24 also did not persist in weeks 36 and 60 (interaction week: week 12: coefficient=–0.194, *P*=.02; week 24: coefficient=–0.221, *P*=.01; week 36: coefficient=–0.16, *P*=.08; week 60: coefficient=–0.17, *P*=.06; Model 0 in Multimedia Appendix 5). A tendency for a stronger reduction of the HADS-A in the intervention group compared with the control group was assessed at week 60 (interaction week x intervention: week 60: coefficient=–0.24, *P*=.06; Model 0 in Multimedia Appendix 5; Figure 3).

A significant reduction in the DLQI was observed at weeks 24 and 60 in all patients but not in weeks 12 and 36 (interaction week: week 12: coefficient=–0.24, *P*=.08; week 24: coefficient=–0.37, *P*=.007; week 36: coefficient=–0.26, *P*=.07; week 60: coefficient=–0.56, *P*<.001; Model 0 in Multimedia Appendix 5). There were no significant differences assessed between the study groups (Model 0 in Multimedia Appendix 5).

In both study groups, the mood of the patients with psoriasis was ameliorated. Compared with scores in the baseline visit, significantly lower scores on the Likert scale were found in all the following visits in all patients (interaction week: week 12: coefficient=–1.80, *P*=.006; week 24: coefficient=–2.12, *P*=.001; week 36: coefficient=–1.83, *P*=.009; week 60: coefficient=–2.64, *P*<.001; Model 0 in Table 3). However, group membership had no significant effect (Model 0 in Table 3).

**Table 3:** Characteristics of the study cohort divided by app use frequency of more or less than 20% at week 60 (N=38).^a^

Characteristics week 60			Overall		App use frequency <20% (n=15)		App use frequency ≥20% (n=18)		*P* value
**Sex, n (%)**									
	Female	15 (39)		5 (33)		9 (50)		.50^b^	
	Male	23 (61)		10 (67)		9 (50)		.50^b^	
**Age (years)**									
	Mean (SD)	46.9 (12.3)		45.0 (14.1)		47.7 (10.8)		.99^c^	
	Median (IQR)	48.0 (39-54)		47.0 (28-53)		49.0 (40-54)		.99^c^	
**BMI^d^** **(kg/m^2^)**									
	Mean (SD)	27.6 (4.7)		27.5 (5.1)		27.7 (4.4)		.99^c^	
	Median (IQR)	26.0 (24.2-32.5)		27.5 (22.5-32.5)		26.0 (24.8-32.7)		.99^c^	
**Alcohol (days/week)**									
	Mean (SD)	1.3 (1.7)		1.5 (1.3)		1.2 (1.8)		.99^c^	
	Median (IQR)	1.0 (0-2.0)		1.0 (1-2.0)		1.0 (0-1)		.99^c^	
Smoker, n (%)			10 (26.3)		2 (13.3)		5 (27.8)		.13^b^
**Employed, n (%)**									
	Yes	34 (89)		13 (87)		17 (94)		.15^b^	
	No	3 (8)		1 (7)		1 (6)		.15^b^	
	Retired	1 (3)		1 (7)		0 (0)		.15^b^	
**Graduation level, n (%)**									
	No final school examination	1 (3)		0 (0)		1 (6)		.23^b^	
	Final school examination after 9 years^e^	5 (13)		1 (7)		3 (17)		.23^b^	
	Final school examination after 10 years^f^	9 (24)		4 (27)		4 (22)		.23^b^	
	Vocational diploma^g^	4 (11)		1 (7)		2 (11)		.23^b^	
	General qualification for university entrance^h^	5 (13)		2 (13)		2 (11)		.23^b^	
	University degree	14 (37)		7 (47)		6 (33)		.23^b^	
**Duration of psoriasis (years)**									
	Mean (SD)	17.1 (13.1)		19.7 (14.3)		14.3 (12.1)		.91^c^	
	Median (IQR)	13.5 (6-24)		23.0 (6-28)		9.0 (6-20)		.91^c^	
Psoriatic arthritis, n (%)			15 (39.5)		8 (53.3)		6 (35.3)		.49^b^
**Antipsoriatic therapy, n (%)**									
	Topical or UV therapy	11 (29)		2 (13)		7 (39)		.12^b^	
	DMARDS^i^	4 (11)		2 (13)		0 (0)		.12^b^	
	Others	2 (5)		1 (7)		1 (6)		.12^b^	
	Biologicals	21 (55)		10 (67)		10 (56)		.12^b^	
	No therapy	0 (0)		0 (0)		0 (0)		.12^b^	
**HADS-D^j^** **(range 0-21)**									
	Mean (SD)	4.0 (4.5)		2.3 (3.4)		5.4 (5.2)		.15^c^	
	Median (IQR)	2.0 (0-7)		1.0 (0-3)		4.0 (1-9)		.15^c^	
**HADS-A^k^** **(range 0-21)**									
	Mean (SD)	4.9 (3.5)		4.2 (3.1)		5.6 (3.5)		.83^c^	
	Median (IQR)	4.0 (2-7)		4.0 (1-6)		5.0 (3-7)		.83^c^	
**DLQI^l^** **(range 0-30)**									
	Mean (SD)	4.4 (5.5)		3.4 (4.9)		5.4 (6.2)		.70^c^	
	Median (IQR)	1.0 (1-8)		1.0 (1-6)		3.0 (1-9)		.70^c^	
**Mood (range 0-3)**									
	Mean (SD)	0.6 (0.8)		0.5 (0.6)		0.8 (0.8)		.61^c^	
	Median (IQR)	0.5 (0-1)		0.0 (0-1)		1.0 (0-1)		.61^c^	
**Daily activity (range 0-30)**									
	Mean (SD)	0.5 (0.7)		0.4 (0.6)		0.7 (0.8)		.63^c^	
	Median (IQR)	0.0 (0-1)		0.0 (0-1)		1.0 (0-1)		.63^c^	
**PASI^m^** **(range 0-72)**									
	Mean (SD)	2.7 (3.3)		2.3 (3.7)		2.5 (2.6)		.99^c^	
	Median (IQR)	1.4 (0.6-3.4)		1.2 (0.2-1.5)		1.85 (0.6-3.4)		.99^c^	
**Pain (range 0-10)^d^**									
	Mean (SD)	1.0 (2.0)		0.8 (1.2)		1.6 (2.6)		.99^c^	
	Median (IQR)	0.0 (0-1)		0.0 (0-2)		0.5 (0-2)		.99^c^	
**Pruritus (range 0-10)**									
	Mean (SD)	1.8 (2.2)		2.1 (2.7)		1.9 (2.0)		.99^c^	
	Median (IQR)	1.0 (0-3)		1.0 (0-3)		1.5 (0-3)		.99^c^	

^a^Data for sex, age, BMI, smoking, alcohol consumption, employment status, graduation level, psoriasis duration, psoriasis arthritis, and therapy were collected at week 0 only.

^b^Categorical variables were analyzed using the chi-square test.

^c^Continuous variables were analyzed using the Bonferroni-test.

^d^For 1 patient, data for weight and height were missing, and for 1 patient, data for pain was missing at week 60. In the intervention group, 5 patients did not download the app at all. Therefore, they could not be divided into the app use frequency subgroups.

^e^Lowest Certificate of Secondary Education in Germany.

^f^Roughly equal to the General Certificate of Secondary Education (no university entrance qualification).

^g^Final school examination after 12 years in combination with vocational education (university entrance qualification for specific degrees only).

^h^Final school examination after 13 years.

^i^DMARD: disease-modifying antirheumatic drug.

^j^HADS-D: Hospital Anxiety and Depression Scale–Depression.

^k^HADS-A: Hospital Anxiety and Depression Scale–Anxiety.

^l^DLQI: Dermatology Life Quality Index.

^m^PASI: Psoriasis Area and Severity Index.

Likewise, a significant reduction in the impairment of daily activities on the Likert scale was found in all follow-up visits compared with baseline visits in both groups, with no significant difference between the study groups (interaction week: week 12: coefficient=–0.51, *P*<.001; week 24: coefficient=–0.53, *P*<.001; week 36: coefficient=–0.55, *P*<.001; week 60: coefficient=–0.68, *P*<.001; Model 0 in Table 3).

**Figure 4:**
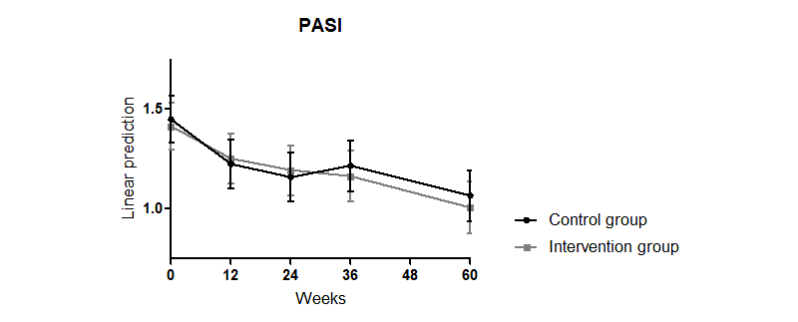
Effects of an educational program combined with a disease management psoriasis app on the PASI score over 60 weeks. PASI: Psoriasis Area and Severity Index.

There was also a significant reduction in the PASI scores observed over the 60 weeks compared with those at week 0 in all patients (interaction week: week 12: coefficient=–0.24, *P*=.04; week 24: coefficient=–0.29, *P*=.008; week 26: coefficient=–0.23, *P*=.04; week 60: coefficient=–0.38, *P*=.001; Model 0 in Table 3). Group membership had no significant effect (Model 0 in Table 3; Figure 4).

A significant reduction in pruritus was assessed in the control and intervention groups at week 60 compared with that at baseline, with no significant difference between the 2 groups (interaction week: week 60: coefficient=–0.97, *P*=.02; Model 0 in Table 3).

No effects were found on the numerical rating scale for skin pain (Model 0 in Table 3).

These findings did not differ if adjusted for sex, age, and disease duration (matching coefficients and *P* values under Model 1 in Table 3).

### App Use Frequency Subgroup Analysis

The intervention group was divided into 2 groups, with patients using the app more or less frequently than 20% (equals one-time use every 5 weeks). Both groups showed similar characteristics (Table 3). Neither demographic nor socioeconomic or disease-related characteristics had any influence on app use frequency.

**Figure 5:**
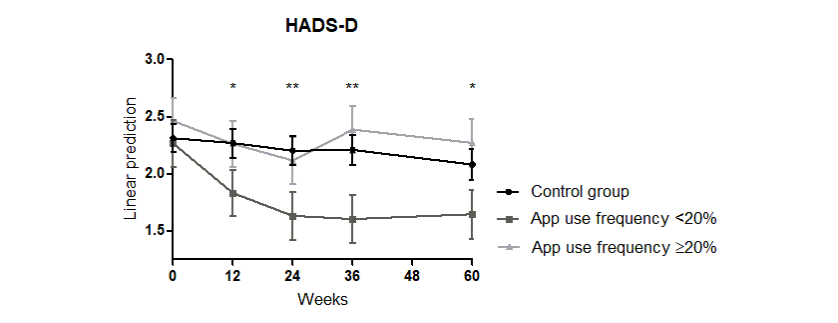
Significant improvement of the HADS-D score in patients with psoriasis using the psoriasis app less than once every 5 weeks (<20%) compared with the control group over 60 weeks. HADS-D: Hospital Anxiety and Depression Scale–Depression. *P≤.05, **P≤.01.

The significant reduction in the HADS-D at weeks 12 and 24 persisted until week 60 in patients, with an app use frequency <20% compared with that of the control group (interaction week x <20%: week 12: coefficient=–0.39, *P*=.03; week 24: coefficient=–0.53, *P*=.004; week 36: coefficient=–0.56, *P*=.004; week 60: coefficient=–0.39, *P*=.04; Model 0 in Multimedia Appendix 6; Figure 5).

**Figure 6:**
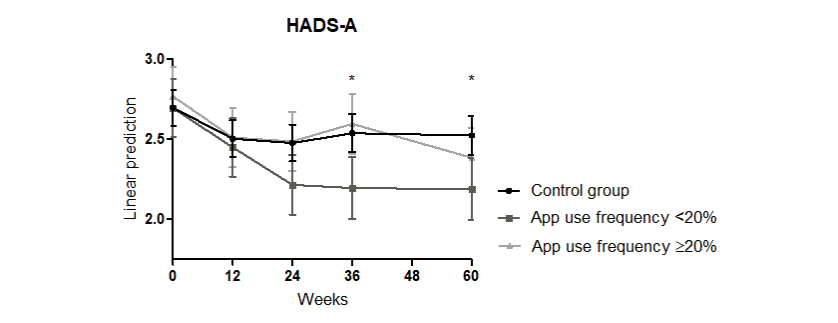
Significant improvement of the HADS-D score in patients with psoriasis using the psoriasis app less than once every 5 weeks (<20%) compared with the control group over 60 weeks. HADS-D: Hospital Anxiety and Depression Scale–Depression. *P≤.05.

Furthermore, patients with an app use frequency of <20% showed a significant reduction in the HADS-A at weeks 36 and 60 (interaction week x <20%: week 36: coefficient=–0.34; *P*=.04; week 60: coefficient=–0.33; *P*=.05; Model 0 in Multimedia Appendix 6; Figure 6).

In patients using the app >20%, no significant reduction in the HADS-D/-A was observed over the 60-week study period (Model 0 in Multimedia Appendix 6; Figures 5 and 6).

**Figure 7:**
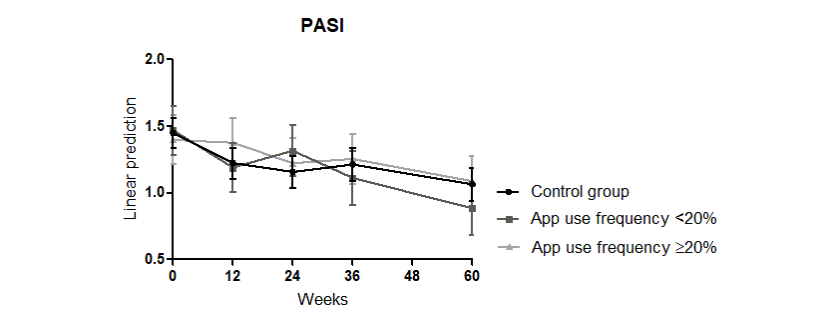
Effects of an educational program combined with a disease management psoriasis app on the PASI score of the subgroups using the psoriasis app more (≥20%) or less (<20%) than once every 5 weeks. PASI: Psoriasis Area and Severity Index.

For the DLQI, mood, daily activity, PASI, and skin pruritus and pain, no differences in the subgroup analysis were found compared with the analysis of the whole group (Model 0 in Multimedia Appendix 6; PASI in Figure 7). These results were independent of sex, age, and disease duration (matching coefficients and *P* values in Model in Multimedia Appendix 6).

## Discussion

### Principal Findings

In this study, we analyzed the effects of an educational program combined with an eHealth smartphone app on the clinical outcomes of patients with psoriasis. We were able to show that this intervention can significantly assist in improving depression and anxiety in patients with psoriasis. Educational programs have proven to be effective in increasing knowledge, quality of life, and self-efficacy in patients with psoriasis in the past [11-14]. To analyze the additional benefits of a disease management app, we tested the educational program applied in this study in a pilot trial first. The trial showed that the educational program per se could lead to a significant improvement in knowledge and self-expertise about the disease and to an amelioration of general health but not to an improvement in patients’ mental health [10]. Therefore, we are convinced that the smartphone app used in this study provides an additional psychological benefit. Improvements in quality of life were also reported by Armstrong et al [23] in an American randomized controlled equivalency trial, in which 296 patients were randomly assigned to either a web-based or in-person care group. eHealth devices such as the one used in this study can probably increase the sense of security in patients by giving them the opportunity to always contact the treating physician using the chat function. In addition, the app might have assisted in building a long-term trusting relationship with the doctor, allowing patients to feel more comfortable with the questions, concerns, and fears about their disease. Furthermore, the combined intervention might have led to a better self-perception of patients’ mental health and disease status by the regular self-report of life quality and mood as well as photodocumentation of the skin. This could have given patients a more structured overview of their disease and thus a greater sense of control. A study by Blome et al [24] showed that greater control is stated as a relevant treatment goal by 92.3% of patients with psoriasis. The fulfillment of this goal can lead to a higher level of satisfaction, which has the potential to reduce depression.

Others have noted that some effects achieved by educational programs vanish after about 6 months [11,13]. In accordance with this, we also observed a significant reduction in the HADS-D score in the intervention group only in the first 6 months. Further studies are needed to determine whether it is possible to lengthen this effect with another educational program after 6 months or by providing regular educational information via the app. Interestingly, a stronger reduction in depression and anxiety levels persisting over the entire 60-week study period was seen in patients using the app less than once every 5 weeks. More frequent app use did not worsen mental health, and there was no further benefit compared with the control group. In accordance with the findings of Ancker et al [25] and Seppen et al [26], we assume that chronically ill patients such as patients with psoriasis do not want to be reminded about their disease too often. New and highly efficient psoriasis treatments such as biologics often lead to lesion-free skin, helping patients feel that they are not sick anymore. Too frequent app use could counteract this effect. We are convinced that an optimal app use frequency needs to be identified for patients. Our data indicate that an app use frequency of approximately once every 5 weeks could be the most beneficial; however, more scientific data are needed.

Web-based care models reduce health care system–related costs and can improve patients’ lives by decreasing the number of clinic visits [23,27,28]. On the patients’ side, this means less absence at work and saved time. Therefore, the implementation of scientifically validated eHealth devices for chronic diseases such as psoriasis seems to be favorable. It will be important to determine how to encourage patients to engage in continuous app use over a long period and whether telemedicine can replace in-person visits or simply support patients' care [19-21,24].

### Limitations

The major limitations of our study are the monocentric design, small study cohort, and limited generalizability of the results. In particular, the number of patients in the subgroups divided by app use frequency was quite low, which could have led to missed or overinterpreted differences between the groups. In addition, the age of our patients was slightly higher than that in other psoriasis studies and differed between the control and intervention groups. The initial PASI score was lower than in other studies, and the percentage of patients treated with systemic therapy was quite high, which could have led to undetected effects. Further studies are necessary to verify our findings on a broader scale and in a multicenter setting.

### Conclusions

In conclusion, the educational program combined with the psoriasis app had a positive impact on the mental health of patients with psoriasis if not used too frequently. The provision of valid and comprehensible knowledge about their own disease and web-based support has the potential to improve health care for patients with psoriasis. Therefore, educating and supporting patients with psoriasis using digital health devices seems to be a promising additional component in the disease management of psoriasis in the long run.

## Appendix

Multimedia Appendix 1 Content overview of the psoriasis educational program.

Multimedia Appendix 2 App design of the study app DermaScope Mobile.

Multimedia Appendix 3 Clinic dashboard of the study app DermaScope Mobile.

Multimedia Appendix 4 CONSORT-EHEALTH (Consolidated Standards of Reporting Trials eHealth) checklist (V 1.6.1).

Multimedia Appendix 5 Random effect regression models over 60 weeks; model 0, unadjusted, and model 1, adjusted for age, sex, and disease duration.

Multimedia Appendix 6 Random effect regression models of the app use frequency subgroups <20% and ≥20% over 60 weeks (N=93; observed=411).

## Abbreviations

DLQI: Dermatology Life Quality Index
HADS-A: Hospital Anxiety and Depression Scale–Anxiety
HADS-D: Hospital Anxiety and Depression Scale–Depression
PASI: Psoriasis Area and Severity Index
