# Supplementary figures and images for: Impact of an eHealth Smartphone App on the Mental Health of Patients With Psoriasis: Prospective Randomized Controlled Intervention Study
Source: JMIR Mhealth Uhealth. 2021 Oct 25;9(10):e28149. doi: 10.2196/28149 (PMC8576562; doi:10.2196/28149)

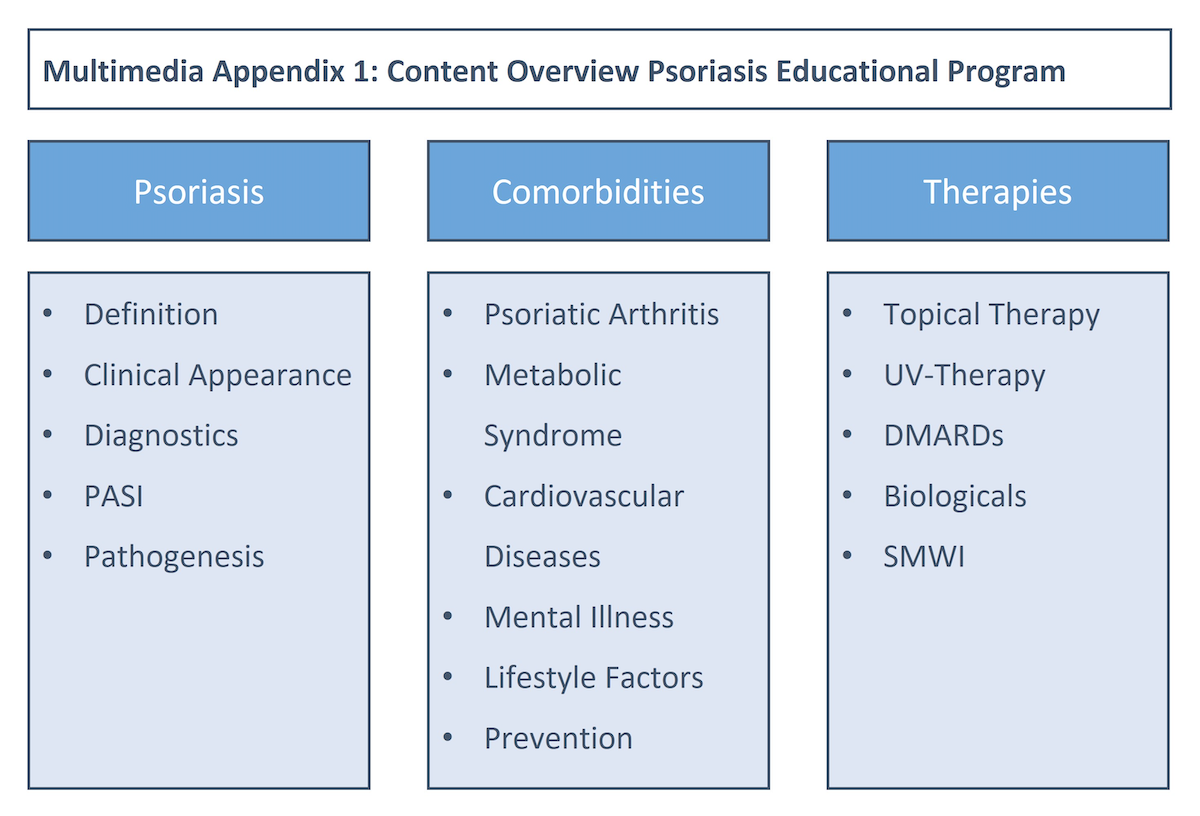

Supplement: Multimedia Appendix 1 [file mhealth_v9i10e28149_app1.png]

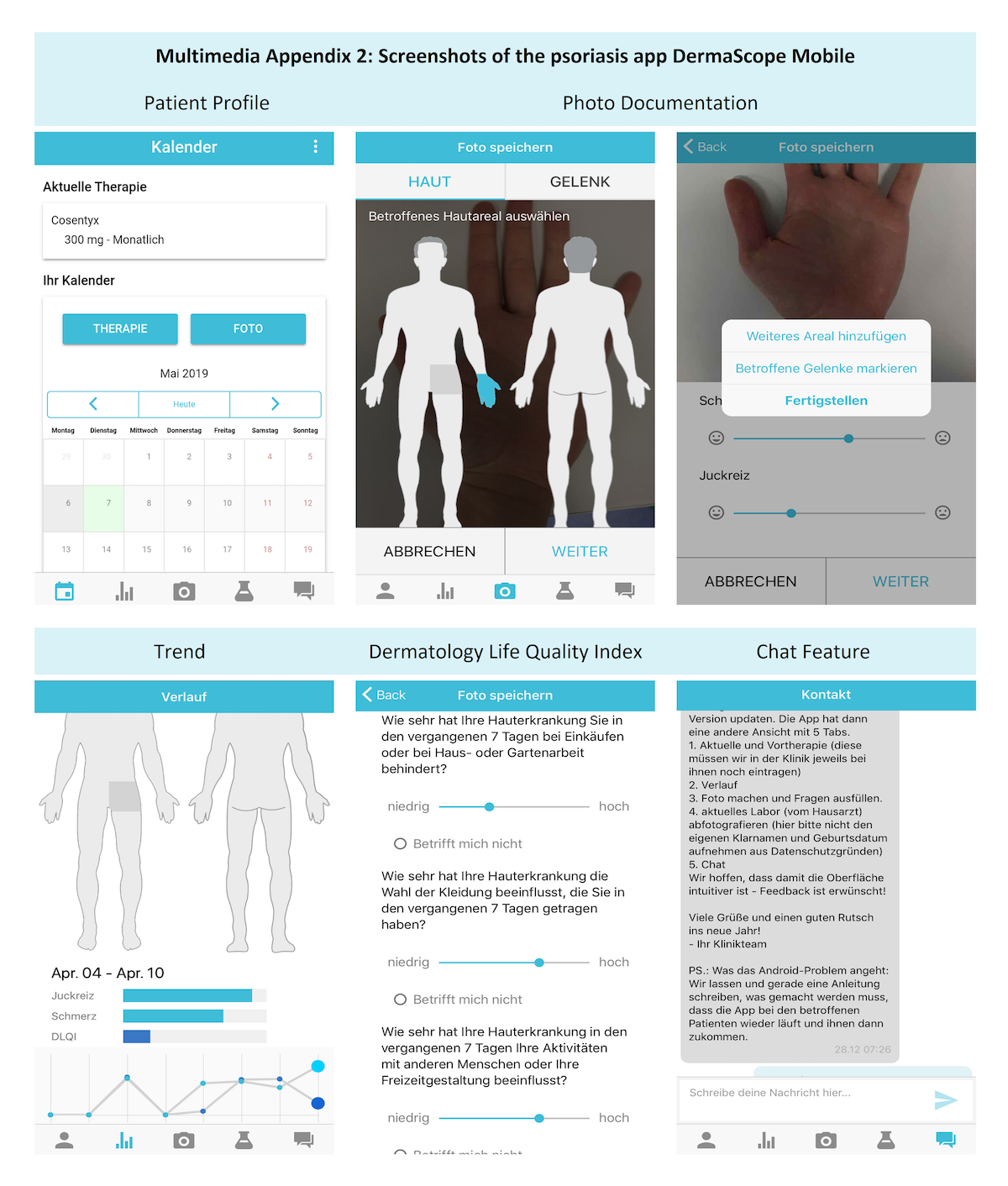

Supplement: Multimedia Appendix 2 [file mhealth_v9i10e28149_app2.png]

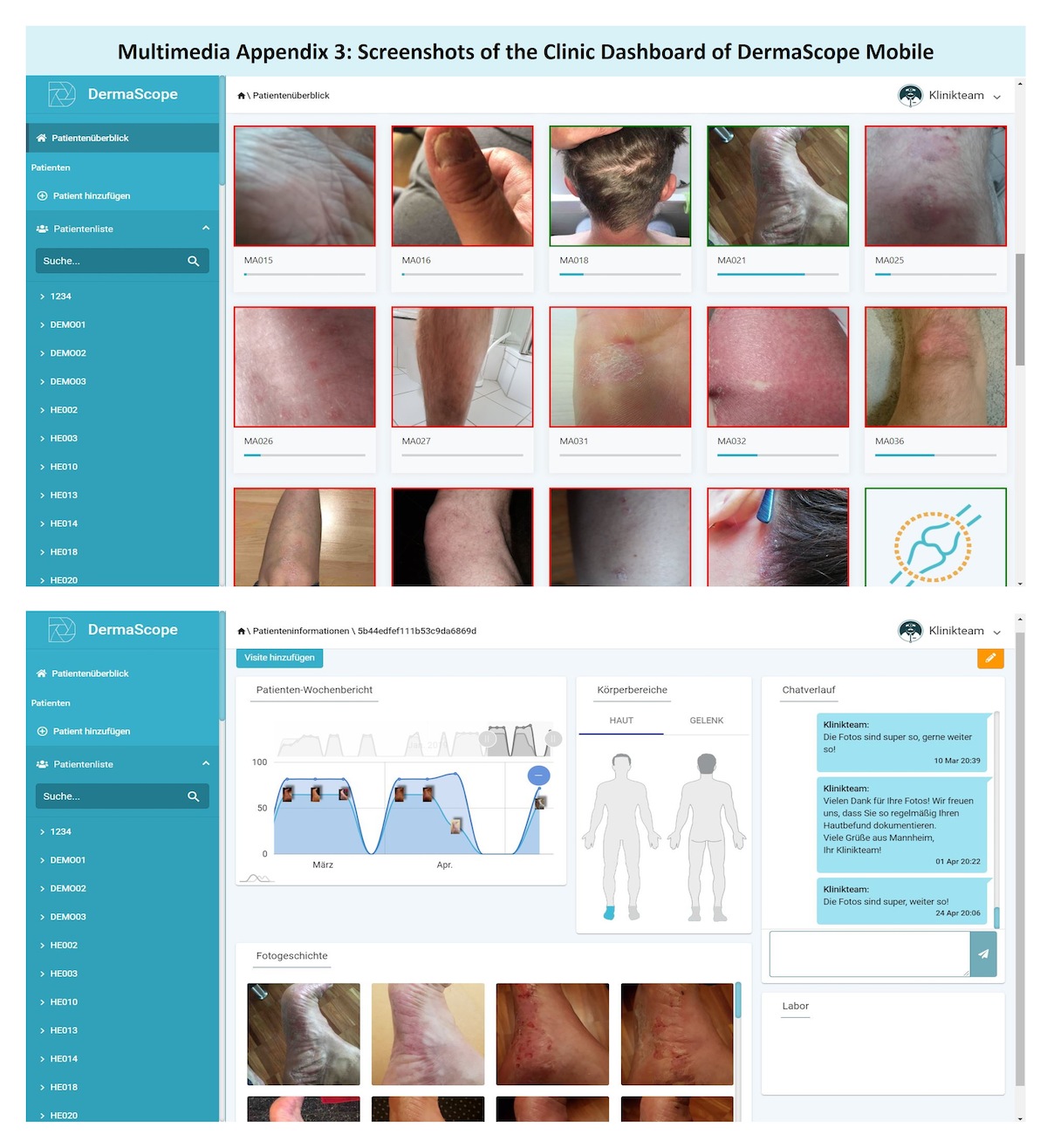

Supplement: Multimedia Appendix 3 [file mhealth_v9i10e28149_app3.png]
