# Supplementary material for: Impact of an eHealth Smartphone App on the Mental Health of Patients With Psoriasis: Prospective Randomized Controlled Intervention Study
Source: JMIR Mhealth Uhealth. 2021 Oct 25;9(10):e28149. doi: 10.2196/28149 (PMC8576562; doi:10.2196/28149)
Supplement: Multimedia Appendix 5 [file mhealth_v9i10e28149_app5.docx]

**Multimedia Appendix 5** Random effect regression models over 60 weeks; model 0 unadjusted and model 1 adjusted for age, sex, and disease duration (N=107; observed=448)^a^.

| Score | | Interaction | Model 0 | | | Model 1 | | |
| --- | --- | --- | --- | --- | --- | --- | --- | --- |
|  | |  | Coefficient | SE | *P* value | Coefficient | SE | *P* value |
|  | |  |  |  |  |  |  |  |
| **HADS-D^b^** | | | | | | | | |
|  | **Week** | | | | | | | |
|  |  | 0 | Reference value | —^c^ | — | Reference value | — | — |
|  |  | 12 | –0.042 | 0.097 | .66 | –0.042 | 0.096 | .66 |
|  |  | 24 | –0.109 | 0.099 | .27 | –0.109 | 0.098 | .27 |
|  |  | 36 | –0.102 | 0.104 | .33 | –0.103 | 0.103 | .32 |
|  |  | 60 | –0.230 | 0.105 | .03 | –0.231 | 0.104 | .03 |
|  | **Week; group** | | | | | | | |
|  |  | 0; control | Reference value | — | — | Reference value | — | — |
|  |  | 12; intervention | –0.289 | 0.140 | .04 | –0.236 | 0.139 | .09 |
|  |  | 24; intervention | –0.397 | 0.143 | .005 | –0.377 | 0.142 | .008 |
|  |  | 36; intervention | –0.231 | 0.148 | .12 | –0.206 | 0.147 | .16 |
|  |  | 60; intervention | –0.150 | 0.150 | .32 | –0.124 | 0.149 | .40 |
| **HADS-A^d^** | | | | | | | | |
|  | **Week** | | | | | | | |
|  |  | 0 | Reference value | — | — | Reference value | — | — |
|  |  | 12 | –0.194 | 0.085 | .02 | –0.192 | 0.084 | .02 |
|  |  | 24 | –0.221 | 0.086 | .01 | –0.218 | 0.085 | .01 |
|  |  | 36 | –0.159 | 0.091 | .08 | –0.157 | 0.090 | .08 |
|  |  | 60 | –0.174 | 0.092 | .06 | –0.172 | 0.090 | .06 |
|  | **Week; group** | | | | | | | |
|  |  | 0; control | Reference value | — | — | Reference value | — | — |
|  |  | 12; intervention | –0.070 | 0.122 | .57 | –0.045 | 0.121 | .71 |
|  |  | 24; intervention | –0.133 | 0.124 | .28 | –0.128 | 0.123 | .30 |
|  |  | 36; intervention | –0.098 | 0.129 | .45 | –0.122 | 0.128 | .34 |
|  |  | 60; intervention | –0.243 | 0.131 | .06 | –0.242 | 0.129 | .06 |
| **DLQI^e^** | | | | | | | | |
|  | **Week** | | | | | | | |
|  |  | 0 | Reference value | — | — | Reference value | — | — |
|  |  | 12 | –0.236 | 0.136 | .08 | –0.239 | 0.136 | .08 |
|  |  | 24 | –0.371 | 0.138 | .007 | –0.374 | 0.138 | .007 |
|  |  | 36 | –0.262 | 0.145 | .07 | –0.261 | 0.145 | .07 |
|  |  | 60 | –0.555 | 0.147 | <.001 | –0.554 | 0.146 | <.001 |
|  | **Week; group** | | | | | | | |
|  |  | 0; control | Reference value | — | — | Reference value | — | — |
|  |  | 12; intervention | –0.137 | 0.196 | .48 | –0.110 | 0.196 | .57 |
|  |  | 24; intervention | –0.037 | 0.199 | .85 | 0.008 | 0.199 | .97 |
|  |  | 36; intervention | –0.071 | 0.206 | .73 | –0.027 | 0.207 | .90 |
|  |  | 60; intervention | –0.057 | 0.209 | .79 | –0.036 | 0.209 | .86 |
| **Mood** | | | | | | | | |
|  | **Week** | | | | | | | |
|  |  | 0 | Reference value | — | — | Reference value | — | — |
|  |  | 12 | –1.797 | 0.655 | .006 | –1.807 | 0.656 | .006 |
|  |  | 24 | –2.116 | 0.664 | .001 | –2.114 | 0.666 | .001 |
|  |  | 36 | –1.830 | 0.700 | .009 | –1.823 | 0.792 | .009 |
|  |  | 60 | –2.637 | 0.706 | <.001 | –2.629 | 0.708 | <.001 |
|  | **Week; group** | | | | | | | |
|  |  | 0; control | Reference value | — | — | Reference value | — | — |
|  |  | 12; intervention | –0.887 | 0.940 | .35 | –0.782 | 0.947 | .41 |
|  |  | 24; intervention | –1.081 | 0.954 | .26 | –0.987 | 0.962 | .31 |
|  |  | 36; intervention | –1.077 | 0.991 | .28 | –0.987 | 0.999 | .32 |
|  |  | 60; intervention | –0.583 | 1.003 | .56 | –0.534 | 1.008 | .60 |
| **Daily activity** | | | | | | | | |
|  | **Week** | | | | | | | |
|  |  | 0 | Reference value | — | — | Reference value | — | — |
|  |  | 12 | –0.508 | 0.146 | <.001 | –0.513 | 0.146 | <.001 |
|  |  | 24 | –0.526 | 0.148 | <.001 | –0.532 | 0.148 | <.001 |
|  |  | 36 | –0.550 | 0.156 | <.001 | –0.552 | 0.156 | <.001 |
|  |  | 60 | –0.682 | 0.157 | <.001 | –0.684 | 0.157 | <.001 |
|  | **Week; group** | | | | | | | |
|  |  | 0; control | Reference value | — | — | Reference value | — | — |
|  |  | 12; intervention | 0.081 | 0.209 | .70 | 0.096 | 0.210 | .65 |
|  |  | 24; intervention | 0.065 | 0.212 | .76 | 0.083 | 0.214 | .70 |
|  |  | 36; intervention | 0.223 | 0.220 | .31 | 0.238 | 0.222 | .28 |
|  |  | 60; intervention | 0.033 | 0.223 | .88 | 0.037 | 0.224 | .87 |
| **PASI^f^** | | | | | | | | |
|  | **Week** | | | | | | | |
|  |  | 0 | Reference value | — | — | Reference value | — | — |
|  |  | 12 | –0.224 | 0.108 | .04 | –0.229 | 0.109 | .04 |
|  |  | 24 | –0.290 | 0.109 | .008 | –0.296 | 0.110 | .007 |
|  |  | 36 | –0.232 | 0.116 | .04 | –0.240 | 0.116 | .04 |
|  |  | 60 | –0.382 | 0.117 | .001 | –0.391 | 0.117 | .001 |
|  | **Week; group** | | | | | | | |
|  |  | 0; control | Reference value | — | — | Reference value | — | — |
|  |  | 12; intervention | 0.062 | 0.155 | .70 | 0.077 | 0.157 | .62 |
|  |  | 24; intervention | 0.070 | 0.158 | .66 | 0.088 | 0.160 | .58 |
|  |  | 36; intervention | –0.018 | 0.163 | .91 | –0.000 | 0.166 | .99 |
|  |  | 60; intervention | –0.026 | 0.166 | .90 | –0.009 | 0.167 | .96 |
| **Pruritus** | | | | | | | | |
|  | Week | 0 | Reference value | — | — | Reference value | — | — |
|  |  | 12 | –0.428 | 0.401 | .29 | –0.430 | 0.401 | .28 |
|  |  | 24 | –0.582 | 0.406 | .15 | –0.578 | 0.407 | .16 |
|  |  | 36 | –0.826 | 0.428 | .05 | –0.811 | 0.429 | .06 |
|  |  | 60 | –0.972 | 0.431 | .02 | 0.954 | 0.432 | .03 |
|  | **Week; group** | | | | | | | |
|  |  | 0; control | Reference value | — | — | Reference value | — | — |
|  |  | 12; intervention | 0.113 | 0.574 | .84 | 0.142 | 0.578 | .81 |
|  |  | 24; intervention | 0.585 | 0.583 | .32 | 0.621 | 0.587 | .29 |
|  |  | 36; intervention | 0.524 | 0.606 | .39 | 0.562 | 0.610 | .36 |
|  |  | 60; intervention | –0.163 | 0.613 | .79 | –0.170 | 0.615 | .78 |
| **Pain** | | | | | | | | |
|  | **Week** | | | | | | | |
|  |  | 0 | Reference value | — | — | Reference value | — | — |
|  |  | 12 | –0.004 | 0.338 | 1.00 | –0.002 | 0.339 | .99 |
|  |  | 24 | 0.272 | 0.343 | .43 | 0.273 | 0.344 | .43 |
|  |  | 36 | 0.360 | 0.362 | .32 | 0.368 | 0.363 | .31 |
|  |  | 60 | 0.015 | 0.364 | .97 | 0.241 | 0.366 | .95 |
|  | **Week; group** | | | | | | | |
|  |  | 0; control | Reference value | — | — | Reference value | — | — |
|  |  | 12; intervention | –0.024 | 0.486 | .96 | –0.021 | 0.490 | .97 |
|  |  | 24; intervention | –0.162 | 0.494 | .74 | –0.157 | 0.498 | .75 |
|  |  | 36; intervention | –0.493 | 0.513 | .34 | –0.501 | 0.518 | .33 |
|  |  | 60; intervention | –0.783 | 0.522 | .13 | –0.797 | 0.525 | .13 |

^a^Model 0 unadjusted; model 1 adjusted for age, sex, and disease duration.

^b^HADS-D: Hospital Anxiety and Depression Scale–Depression.

^c^.

^d^HADS-A: Hospital Anxiety and Depression Scale–Anxiety.

^e^DLQI: Dermatology Life Quality Index.

^f^PASI: Psoriasis Area and Severity Index.
