# Supplementary material for: Impact of an eHealth Smartphone App on the Mental Health of Patients With Psoriasis: Prospective Randomized Controlled Intervention Study
Source: JMIR Mhealth Uhealth. 2021 Oct 25;9(10):e28149. doi: 10.2196/28149 (PMC8576562; doi:10.2196/28149)
Supplement: Multimedia Appendix 6 [file mhealth_v9i10e28149_app6.docx]

**Multimedia Appendix 6.** Random effect regression models of the app use frequency subgroups <20% and ≥20% over 60 weeks (N=93; observed=411).^a^

| Score | | Interaction | Model 0 | | | Model 1 | | |
| --- | --- | --- | --- | --- | --- | --- | --- | --- |
|  | |  | Coefficient | SE | *P* value | Coefficient | SE | *P* value |
|  | |  |  |  |  |  |  |  |
| **HADS-D^b^** | | | | | | | | |
|  | **Week; group** | | | | | | | |
|  |  | 0; control | Reference value | —^c^ | — | Reference value | — | — |
|  |  | 12; <20% | –0.392 | 0.175 | .03 | –0.392 | 0.175 | .03 |
|  |  | 12; ≥20% | –0.160 | 0.178 | .37 | –0.158 | 0.178 | .37 |
|  |  | 24; <20% | –0.525 | 0.180 | .004 | –0.525 | 0.180 | .004 |
|  |  | 24; ≥20% | –0.239 | 0.178 | .18 | –0.237 | 0.178 | .18 |
|  |  | 36; <20% | –0.559 | 0.192 | .004 | –0.558 | 0.192 | .004 |
|  |  | 36; ≥20% | 0.025 | 0.181 | .89 | 0.028 | 0.181 | .88 |
|  |  | 60; <20% | –0.389 | 0.192 | .04 | –0.388 | 0.192 | .04 |
|  |  | 60; ≥20% | 0.037 | 0.184 | .84 | 0.040 | 0.184 | .83 |
| **HADS-A^d^** | | | | | | | | |
|  | **Week; group** | | | | | | | |
|  |  | 0; control | Reference value | — | — | Reference value | — | — |
|  |  | 12; <20% | –0.052 | 0.155 | .74 | –0.053 | 0.155 | .73 |
|  |  | 12; ≥20% | –0.064 | 0.157 | .68 | –0.063 | 0.158 | .69 |
|  |  | 24; <20% | –0.260 | 0.160 | .10 | –0.264 | 0.160 | .10 |
|  |  | 24; ≥20% | –0.062 | 0.158 | .70 | –0.061 | 0.158 | .70 |
|  |  | 36; <20% | –0.341 | 0.170 | .04 | –0.343 | 0.170 | .04 |
|  |  | 36; ≥20% | –0.014 | 0.161 | .93 | –0.012 | 0.161 | .94 |
|  |  | 60; <20% | –0.334 | 0.170 | .05 | –0.336 | 0.170 | .049 |
|  |  | 60; ≥20% | –0.211 | 0.163 | .20 | –0.208 | 0.163 | .20 |
| **DLQI^e^** | | | | | | | | |
|  | **Week; group** | | | | | | | |
|  |  | 0; control | Reference value | — | — | Reference value | — | — |
|  |  | 12; <20% | –0.252 | 0.252 | .32 | –0.251 | 0.252 | .32 |
|  |  | 12; ≥20% | 0.130 | 0.256 | .61 | 0.125 | 0.255 | .62 |
|  |  | 24; <20% | –0.057 | 0.260 | .83 | –0.052 | 0.259 | .84 |
|  |  | 24; ≥20% | 0.215 | 0.257 | .40 | 0.210 | 0.256 | .41 |
|  |  | 36; <20% | 0.017 | 0.275 | .95 | 0.017 | 0.275 | .95 |
|  |  | 36; ≥20% | 0.018 | 0.261 | .95 | 0.008 | 0.260 | .97 |
|  |  | 60; <20% | –0.000 | 0.276 | 1.00 | –0.001 | 0.276 | .99 |
|  |  | 60; ≥20% | 0.038 | 0.265 | .89 | 0.026 | 0.264 | .92 |
| **Mood** | | | | | | | | |
|  | **Week; group** | | | | | | | |
|  |  | 0; control | Reference value | — | — | Reference value | — | — |
|  |  | 12; <20% | –0.684 | 1.252 | .59 | –0.680 | 1.252 | .59 |
|  |  | 12; ≥20% | –1.537 | 1.268 | .23 | –1.551 | 1.268 | .22 |
|  |  | 24; <20% | –0.734 | 1.288 | .57 | 0.715 | 1.288 | .58 |
|  |  | 24; ≥20% | –1.486 | 1.273 | .24 | –1.509 | 1.273 | .24 |
|  |  | 36; <20% | –1.162 | 1.364 | .40 | –1.162 | 1.364 | .40 |
|  |  | 36; ≥20% | –1.432 | 1.293 | .27 | –1.466 | 1.293 | .26 |
|  |  | 60; <20% | –0.219 | 1.368 | .87 | –0.221 | 1.368 | .87 |
|  |  | 60; ≥20% | –1.461 | 1.131 | .27 | –1.502 | 1.311 | .25 |
| **Activity** | | | | | | | | |
|  | **Week; group** | | | | | | | |
|  |  | 0; control | Reference value | — | — | Reference value | — | — |
|  |  | 12; <20% | 0.158 | 0.273 | .56 | 0.162 | 0.272 | .55 |
|  |  | 12; ≥20% | 0.098 | 0.277 | .72 | 0.093 | 0.275 | .73 |
|  |  | 24; <20% | 0.146 | 0.281 | .60 | 0.159 | 0.280 | .57 |
|  |  | 24; ≥20% | 0.063 | 0.278 | .82 | 0.060 | 0.277 | .83 |
|  |  | 36; <20% | 0.323 | 0.298 | .28 | 0.328 | 0.297 | .27 |
|  |  | 36; ≥20% | 0.193 | 0.282 | .49 | 0.184 | 0.281 | .51 |
|  |  | 60; <20% | 0.189 | 0.298 | .53 | 0.193 | 0.297 | .52 |
|  |  | 60; ≥20% | 0.015 | 0.286 | .96 | 0.001 | 0.285 | 1.00 |
| **PASI^f^** | | | | | | | | |
|  | **Week; group** | | | | | | | |
|  |  | 0; control | Reference value | — | — | Reference value | — | — |
|  |  | 12; <20% | –0.055 | 0.205 | .79 | –0.050 | 0.205 | .81 |
|  |  | 12; ≥20% | 0.204 | 0.208 | .33 | 0.210 | 0.208 | .31 |
|  |  | 24; <20% | 0.137 | 0.211 | .52 | 0.150 | 0.211 | .48 |
|  |  | 24; ≥20% | 0.116 | 0.208 | .58 | 0.123 | 0.209 | .56 |
|  |  | 36; <20% | 0.121 | 0.224 | .59 | 0.108 | 0.224 | .63 |
|  |  | 36; ≥20% | 0.094 | 0.212 | .66 | 0.102 | 0.212 | .63 |
|  |  | 60; <20% | 0.200 | 0.224 | .37 | –0.187 | 0.225 | .41 |
|  |  | 60; ≥20% | 0.077 | 0.215 | .72 | 0.086 | 0.215 | .69 |
| **Pruritus** | | | | | | | | |
|  | **Week; group** | | | | | | | |
|  |  | 0; control | Reference value | — | — | Reference value | — | — |
|  |  | 12; <20% | 0.271 | 0.750 | .72 | 0.271 | 0.748 | .72 |
|  |  | 12; ≥20% | –0.098 | 0.760 | .90 | 0.117 | 0.758 | .88 |
|  |  | 24; <20% | 0.853 | 0.772 | .27 | 0.860 | 0.770 | .26 |
|  |  | 24; ≥20% | 0.681 | 0.763 | .37 | 0.655 | 0.761 | .39 |
|  |  | 36; <20% | –0.102 | 0.818 | .90 | –0.113 | 0.816 | .89 |
|  |  | 36; ≥20% | 1.012 | 0.775 | .19 | 0.975 | 0.773 | .21 |
|  |  | 60; <20% | 0.044 | 0.820 | .96 | 0.032 | 0.818 | .97 |
|  |  | 60; ≥20% | –0.268 | 0.785 | .73 | 0.315 | 0.784 | .69 |
| **Pain** | | | | | | | | |
|  | **Week; group** | | | | | | | |
|  |  | 0; control | Reference value | — | — | Reference value | — | — |
|  |  | 12; <20% | –0.346 | 0.640 | .59 | –0.349 | 0.640 | .59 |
|  |  | 12; ≥20% | 0.375 | 0.650 | .56 | 0.366 | 0.649 | .57 |
|  |  | 24; <20% | –0.386 | 0.660 | .56 | –0.393 | 0.660 | .55 |
|  |  | 24; ≥20% | 0.415 | 0.653 | .52 | 0.407 | 0.652 | .53 |
|  |  | 36; <20% | –0.758 | 0.701 | .28 | –0.771 | 0.700 | .27 |
|  |  | 36; ≥20% | –0.199 | 0.663 | .76 | –0.213 | 0.663 | .75 |
|  |  | 60; <20% | –0.624 | 0.714 | .38 | –0.635 | 0.714 | .37 |
|  |  | 60; ≥20% | –0.674 | 0.673 | .32 | –0.695 | 0.673 | .30 |

^a^Model 0 unadjusted; Model 1 adjusted for age, sex, and disease duration.

^b^HADS-D: Hospital Anxiety and Depression Scale- Depression.

^c^.

^d^ HADS-A: Hospital Anxiety and Depression Scale- Anxiety.

^e^ DLQI: Dermatology Life Quality Index.

^f^ PASI: Psoriasis Area and Severity Index.
